# Supplementary material for: Chromothripsis during telomere crisis is independent of NHEJ, and consistent with a replicative origin
Source: Genome Res. 2019 May;29(5):737–49. doi: 10.1101/gr.240705.118 (PMC6499312; doi:10.1101/gr.240705.118)
Supplement: Supplemental Material [file supp_gr.240705.118_Supplemental_file_1.zip › contigs/annotated_contigs/DB48/contig.3.DB48_length_621_mean_cov_7.18035426731.docx]

**DB48_length_621_mean_cov_7.18035426731**

```
CTTGAGGTCAGGAGTTCAAGACCAGTCTGGTCAACATGGTGAAACCCTGTCTCTACTAAAAATACAAAAAATTAGCCAGGCATGGTGGC
 >chr3:51473788-51473935 + E=1e-49 p=0e+00
GGGCA|CCTGTAATCCCAGCTACTCGGGAGACTGAGGCAGAAGAATTGCTTGAACCAGG|GAGGCGCAGGTAGCAGTGAGCCAAGATAG
      >chr13:54750310-54750614 + E=3e-168
CACCGCTGCACTCCAGCCTGGGTGACCAGAGCAAGACTTTGTCTCAAAAAAACAAACAAAACAACACAAAAAAACTAGAGATGGGAATA

ACAGATTCTCAGGGAGAGGATTGCCAAGTGTGTTACCTATAAGGTTTTAAAATGCTTCTCATGAGATTTTTGTATTTCTCCCTTTATCT

TCCTTCCACTGCATTCTCATTGAGGAACACCATTCTCACTGA|AT|TAAATAAAGATCCAGATTTTGTCAATAGTGTGTTTAATTAAGC
                                           >chr13:54752083-54752308 + E=1e-123
AGACCCAATAAGAGCATCAATAAGCTCTTGAAAGAAGCCTAGATACGGAGAAACTTACGGAGAGCTAATATGAAAGGTAGGTAAATGAC

TTAAGCCTATGGTGGGCTATTTCAGAAGACTGGCTCCAGATGCATGGCCACTGTCTCATTAAACTATGTTGTATTCAAGGCATGCCATC

TT
```
